# An FPGA-Based ECU for Remote Reconfiguration in Automotive Systems

**DOI:** 10.3390/mi12111309

**Published:** 2021-10-26

**Authors:** Kwonneung Cho, Jeongeun Kim, Do Young Choi, Young Hyun Yoon, Jung Hwan Oh, Seung Eun Lee

**Affiliations:** Department of Electronic Engineering, Seoul National University of Science and Technology, Seoul 01811, Korea; chokwonneung@seoultech.ac.kr (K.C.); kimjeongeun@seoultech.ac.kr (J.K.); choidoyoung@seoultech.ac.kr (D.Y.C.); yoonyounghyun@seoultech.ac.kr (Y.H.Y.); ohjunghwan@seoultech.ac.kr (J.H.O.)

**Keywords:** automotive systems, electronic control units, hardware-reconfigurable ECUs, in-system reconfiguration, Zipwire controller

## Abstract

Growing interest in intelligent vehicles is leading automotive systems to include numerous electronic control units (ECUs) inside. As a result, efficient implementation and management of automotive systems is gaining importance. Flexible updating and reconfiguration of ECUs is one appropriate strategy for these goals. Software updates to the ECUs are expected to improve performance and bug handling, but there are limitations due to the fixed hardware circuit. By applying hardware-reconfigurable ECUs to the automotive system, patches that are not able to be handled with only software updates are enabled. In this paper, a remotely hardware-reconfigurable ECU for automotive systems is proposed. The proposed ECU is implemented with a field programmable gate array (FPGA) and microcontroller unit (MCU) to support in-system reconfiguration (ISR). The communication interface between the FPGA and MCU employs Zipwire communication for high speed and resilient communication. For the Zipwire communication, a Zipwire controller is designed and implemented in the FPGA. The proposed hardware-reconfigurable ECU was successfully implemented, and feasibility was demonstrated.

## 1. Introduction

As interest in intelligent vehicles is increasing, automotive systems will include a number of electronic control units (ECUs) in order to provide various functions and controls [1]. Not only high-end cars, but also entry models, are planning to be equipped with high performance systems with multiple ECUs. Considering high-end cars are already applying more than 100 ECUs and embedded computing units each, it can be anticipated that there will be more demand for highly efficient systems for vehicles [2,3,4]. In addition, self-driving vehicles and modern automobiles employ multi-modal sensors, numerous digital processors, complex embedded software, and multiple in-vehicle networks [5,6,7,8]. Since the automotive system needs to perform various tasks, such as dealing with amounts of sensor data and reliable communication with electrical units, the system becomes more complex and larger, including numerous ECUs inside [9,10]. As a result, there are some challenges for implementing and managing the automotive systems, and flexible updating and reconfiguration of ECUs are required.

The ECU that controls core applications in the automotive system implements various functions which are initially programmed when the ECU is rolled out. The initial program occasionally needs to be updated for various reasons, such as performance improvement, security enhancement and error handling [11,12,13,14,15]. In order to update the firmware of the ECU with convenience, the techniques for automotive wireless software updates are under study, achieving efficient and secure goals with over the air (OTA) software updates [12,16,17]. However, the software update only changes the functional operation of an ECU which has specific hardware circuits. Therefore, there are limitations to the reconfiguration domain and performance improvement.

With a hardware reconfiguration of an ECU, there are some advantages that hard to achieve with software updates in the automotive system. For instance, when implementing communication networks in automotive systems, hardware-reconfigurable ECUs improve compatibility and performance. In automotive communication, the controller area network (CAN) protocol is widely adopted due to its high reliability [18,19]. However, as the number of ECUs increase, limited bandwidth makes it difficult to implement real-time communication [2,18]. In order to address the issue, a CAN with flexible data rate (CAN FD) protocol supports wider bandwidths while maintaining the characteristics of a CAN protocol such as arbitration, stuffing, and acknowledge frame [19,20,21]. In addition, the in-vehicle network, such as local interconnect network (LIN), FlexRay, and single edge nibble transmission (SENT) also can be revised to satisfy the demands of bandwidth and reliability, as the number of ECUs adopted by automobiles are increasing [22]. In that case, it is hard to replace the revised network with software updates, but hardware reconfiguration enables the ECU to support the network as long as the resource of the ECU is suitable to implement the revised protocol. In this point of view, hardware-reconfigurable ECUs in automotive systems save the cost of updating the network and provide up-to-date features for passengers.

In order to apply a reconfigurable ECU, employing a field programmable gate array (FPGA) is appropriate due to the flexible circuit configuration [23,24,25,26]. By applying the FPGA-based ECU, automotive systems are able to achieve performance improvements which are hard to achieve with only software updates. FPGA-based ECUs provide hardware flexibility for functional extension, physical specification of in-vehicle networks, and the implementation of multiple ECUs on one chip. Several studies have been conducted applying the configuration characteristics of the FPGA in automotive systems and industries [1,2,27]. In [1], the authors proposed ECU architecture for secure and dependable automotive system with FPGA implementation. In [2], the authors designed an extensible FlexRay communication controller for FPGA-based automotive systems. The authors of [27] presented the technology of secure boot and OTA updates for a reconfigurable hardware of internet of things (IoT) device which is implemented on the FPGA. When it comes to reconfiguring the FPGA-based ECU in an automotive system, the updating process needs to be progressed in specific place, incurs a high cost, and is time-consuming [28,29]. As reconfiguring the FPGA-based ECU with physical lines is inconvenient and risky due to the complex structure of automobiles, a convenient way for the remote reconfiguration is required.

In this paper, a hardware reconfigurable ECU with in-system reconfiguration (ISR) is proposed. In order to address the inconvenience of ECU updates, an FPGA-based ISR that is able to apply remote hardware updates is presented. The proposed reconfiguration of the ECU is implemented with FPGA and microcontroller unit (MCU) co-designed architecture. The MCU is employed to implement the ISR and to control the functionality of the FPGA. For communication with the MCU and FPGA, Zipwire communication is adopted because of its reliability and high-speed serial communication [30]. The ECUs ] implemented on the FPGA and its reconfiguration process are controlled by the MCU through Zipwire communication for system stability. By applying an MCU, FPGA and Zipwire communication, the proposed ISR for a hardware-reconfigurable ECU is successfully implemented.

The contributions of this paper are as follows. The architecture for the reconfigurable ECU is presented and implemented on Intel MAX10 FPGA and NXP MPC5777C automotive MCU. Detailed methods of the in-system reconfiguration are described with error sources and solutions that affect system stability. A Zipwire controller is designed with Verilog HDL to provide Zipwire interface to the proposed ECU. The designed Zipwire controller is implemented on the MAX10 FPGA with less than 1% resource utilization and verified by experimenting with the NXP MCU.

The remainder of this paper is organized as follows. In Section 2, the backgrounds for Zipwire communication are described. The description of in-system programming is also presented for FPGA configuration flow. Section 3 presents the proposed system architecture and mechanism. Section 4 explains the result and discussion. Finally, the conclusion is given in Section 5.

## 2. Background

### 2.1. Zipwire Communication

The Zipwire protocol was invented for inter-processor communication in automotive systems [30]. Zipwire, a master–slave-based full duplex, is point to point communication that consists of five pins. Since Zipwire adopts low voltage differential signal (LVDS), the two pairs of positive and negative pins are used for transmission, and one other pin is a reference clock pin. The reference clock is generated by a slave node and provided to a master node. The master node can select the reference clock or oscillator clock source as an input of internal phase-locked loop (PLL) to operate the Zipwire module. The Zipwire frame includes several frame units such as request frame or response frame to communicate between the master and slave node. Every frame is generated by the LVDS fast asynchronous serial transmission interface (LFAST) and serial inter-processor interface (SIPI) modules.

**Figure 1:**
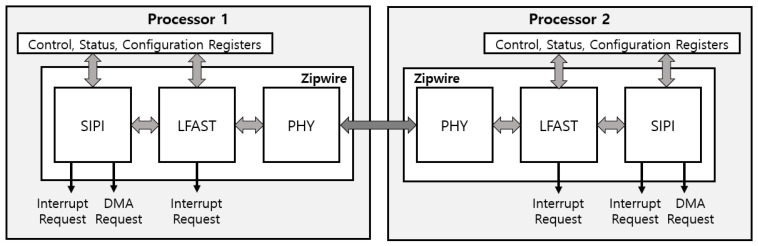
Zipwire communication overview.

The Zipwire module consists of the physical layer module, LFAST module, and SIPI module, as shown in Figure 1. The physical module acts as transceiver of the Zipwire module. Therefore, it transforms the whole frame into differential signals. The LFAST module integrates a frame generated from the SIPI module. The master and slave node can synchronize communication and get the information of the whole frame as the LFAST module adds the sync and header field to the frame. The SIPI module builds a payload of the Zipwire frame and informs the transmission ID and data to the target node.

**Figure 2:**
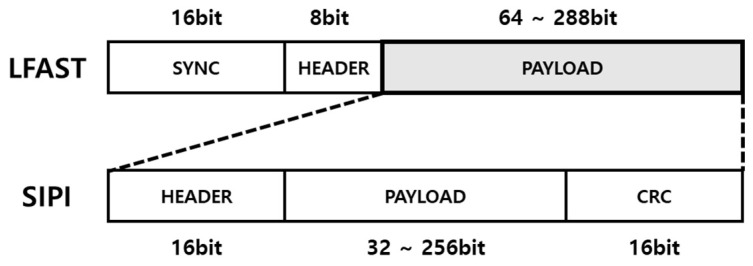
Zipwire protocol.

As shown in Figure 2, the frame is made up of 16-bit sync field, 8-bit header, and 32 to 288-bit payload. The payload of LFAST frame contains the SIPI header, actual payload, and cyclic redundancy check (CRC) of the SIPI. The LFAST sync field which has fixed 16-bit 1010_1000_0100_1011 data, enables synchronized communication between the master and slave node. The LFAST header contains 3-bit payload information, 4-bit data channel information and 1-bit clear to sent (CTS) information. The SIPI header consists of a 3-bit transaction ID from request frame, 5-bit frame command, 3-bit channel number, and 5-bit reserved bits. Since the LFAST frame has several fields, the SIPI payload contains from 32-bit to 256-bit data or address of the frame. The CRC field, calculation results of CRC-16, is used to verify the reliability of frame.

In consideration of communication between a MCU and FPGA, it is essential to ensure high-speed and high reliability in an automotive system. The Zipwire protocol has the advantages of low power consumption and high data rate when adopting the LVDS interface [31]. Additionally, Zipwire can qualify the frame by adopting CRC.

### 2.2. In-System Programming

**Figure 3:**
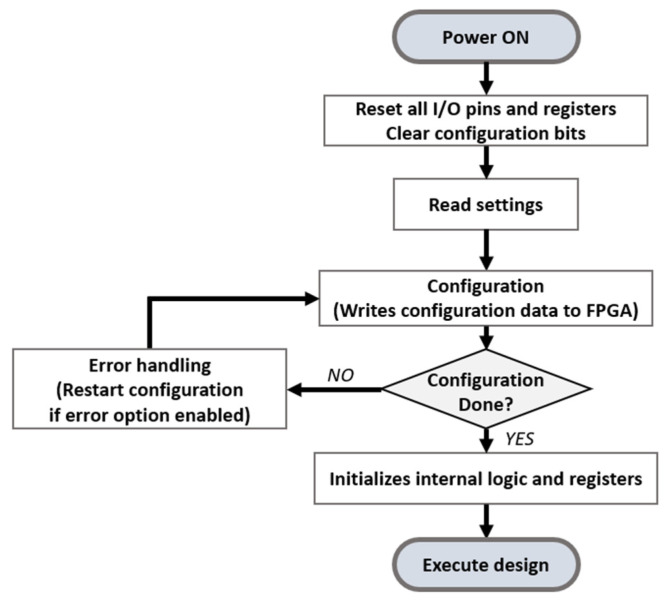
FPGA configuration sequence.

The FPGA offers in-system programming that provides configuration, erasing, and verification [32]. In order to execute a specific logic on an FPGA, the configuration data which includes the information of the logic needing to be loaded to the internal memory of the FPGA, such as configuration RAM or configuration latches [32,33]. Since internal memory is a volatile memory, the configuration data must be stored in flash memory and the data must be shifted to the internal memory when the FPGA powers up. For implementing in-system reconfiguration (ISR), the configuration process of the FPGA needs to be controlled. In Figure 3, the configuration sequence is presented. The FPGA resets IO pins, registers, and clears internal memory to start configuration. The FPGA reads the settings and loads configuration data to the internal memory. During configuration, the FPGA receives instruction, addresses, and data through the external interface that loads the configuration data directly to the configuration RAM, or through the internal programming sequence that shifts the configuration data from the internal flash memory to the configuration RAM. As the various interfaces for configuration are supported according to the FPGA models, employing an appropriate interface is required. When the loading process is completed, the FPGA initializes the internal registers and executes the design. In this work, an Intel MAX 10 FPGA that provides internal configuration flash memory (CFM) for the internal programming is employed.

## 3. System Architecture and Mechanism

### 3.1. Overall Design of System

**Figure 4:**
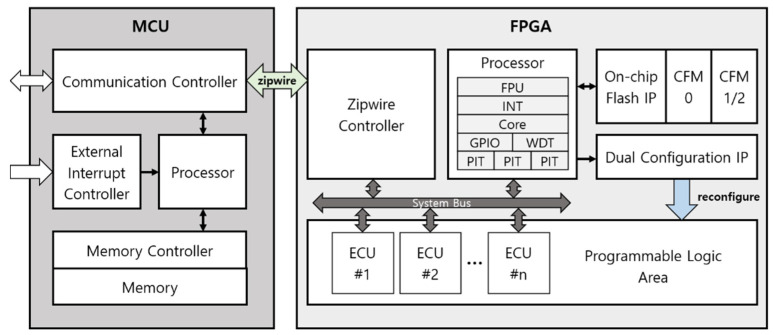
Architecture of the proposed system.

The proposed system is composed of FPGA-based ECUs and the MCU performing the ISR. Figure 4 shows the overall architecture of the proposed design. The MCU communicates with the external system, and stores reconfiguration data including the ECU logics in the memory. The external system can be an on-board diagnostic (OBD), which is generally utilized in automotive applications, or server sending system that employs OBD-2 protocols or 5G to implement the remote ISR. The MCU and the FPGA communicate with Zipwire communication that ensures high speed and stable data transfers. The FPGA includes internal flash memories and a programmable logic (PL) area. The PL area implements the ECUs’ logic and enables the ECUs to achieve more diverse operations.

The MCU contains the communication controller, processor, external interrupt controller, memory controller, and memory. The MCU receives the reconfiguration data from the external system through the communication controller and stores the data in the memory. The external interrupt controller receives commands from the external system and initiates the reconfiguration process. The memory controller handles memory storing and according to command from the external system, the MCU transmits reconfiguration data and triggers to the FPGA.

In the FPGA, the Zipwire controller, processor, on-chip flash IP, configuration flash memories (CFMs) and dual config IP are implemented for reconfigurable ECUs. The processor includes peripherals such as a floating point unit (FPU), interrupt controller, GPIO, watchdog timer (WDT), and programmable interval timers (PITs). Through the peripherals, the processor controls the ECUs and operates functions for the automotive system. The on-chip flash IP and dual configuration IP are connected to the processor. The processor sets up registers of the on-chip flash IP and dual configuration IP to initiate the FPGA reconfiguration. In order to reconfigure the FPGA logic, the FPGA receives the reconfiguration data and the trigger from the MCU. The on-chip flash IP stores the reconfiguration data in CFM0 or CFM1/2. When the storing data is completed, the processor controls the dual configuration IP to select the target logic of CFM0 or CFM1/2. The dual configuration IP updates the configuration RAM with the selected reconfiguration data. Consequently, the PL area is reconfigured with the target logic, and the design is executed. Since the reconfiguration data are stored in the CFM but the current logic is executed in configuration RAM, the FPGA-based ECU can be reconfigured during run-time.

### 3.2. Flow of In-System Reconfiguration

**Figure 5:**
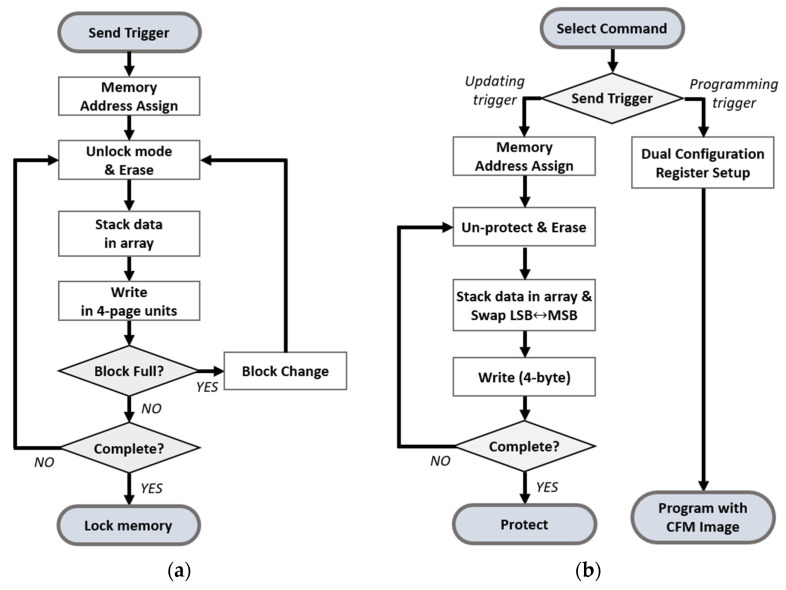
(**a**) Data storing flow in the MCU. (**b**) Reconfiguration flow in the FPGA.

The flow of storing data from the external system to the MCU is demonstrated in Figure 5a. When the external system sends a trigger to the MCU, the MCU assigns the memory address to store the reconfiguration data. During the transmission, the data are stacked in an array. When the transfer process is completed, the MCU memory is locked and the stable state is confirmed to guarantee memory reliability.

Figure 5b depicts the reconfiguration flow. The MCU sends a trigger to the FPGA according to the received command from an external system. The reconfiguration process of the FPGA is divided into two sequences according to the received trigger. When the FPGA receives updating trigger, the MCU transmits the reconfiguration data to update, and the FPGA stores the data in CFM. In order to update the CFM, the processor in the FPGA allocates the start and end address for the reconfiguration data and executes memory erasing with the on-chip flash IP. After the erasing, the on-chip flash IP writes the reconfiguration data to the CFM considering the bit order and data size of the CFM. When the FPGA receives the programming trigger, the processor sets the registers of the dual configuration IP. The dual configuration IP controls the internal programming sequence and updates the PL area with the received reconfiguration data.

### 3.3. FPGA Reconfiguration Feature

In order to implement the FPGA reconfiguration and revise the CFM area on the FPGA, the Intel dual configuration IP and Nios II processor are employed [32]. When the design to update is compiled, the raw programming data file, programmer object file, and memory map file are generated. The memory map file is required to be checked to verify the address of CFM and modified according to the assigned address of the configuration data. The programmer object file contains the logic of the processor and instruction code data. The code data needs to be loaded to the external flash memory of the FPGA board in order to boot the processor. The raw programming data file includes the actual bit data to configurate the target logic. The reconfiguration process starts with receiving the raw programming data from the external system. The MCU stores the raw data in memory with a 4-page unit and transmits the data to the FPGA with the trigger.

### 3.4. Design of the Zipwire Controller

The Zipwire controller unit is designed for communication between the MCU and processor in the FPGA. In proposed architecture, the MCU controls ECUs through Zipwire communication as a master. The FPGA processor also controls the ECUs, operating with received data from the MCU or sending data about the status of the ECU to the MCU. In addition, the FPGA processor communicates with the Zipwire controller via the system bus.

**Figure 6:**
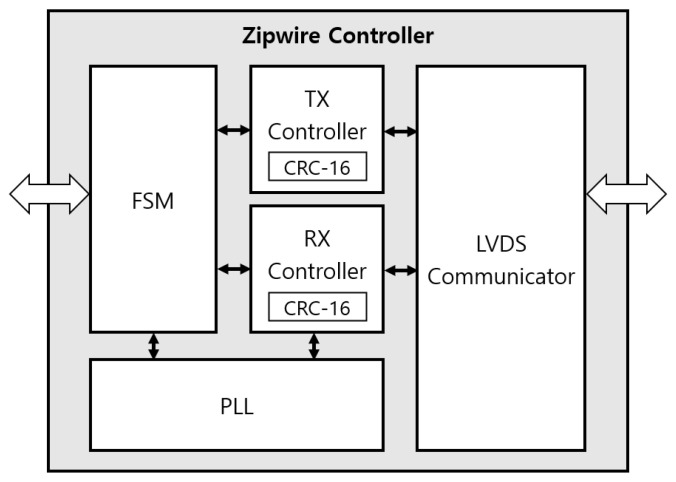
Structure of Zipwire controller.

The Zipwire controller is made up of the FSM, LVDS communicator, Tx controller, Rx controller, and PLL module as shown in Figure 6. The FSM controls overall modules and manages the communication sequence and errors. The LVDS communicator facilitates the LVDS interface which has a noise-resistant characteristic. The Rx controller stores and interprets the Zipwire frame data. The Tx controller sends the received data through the LVDS transmitter. The receiver and the transmitter include a CRC-16 module to detect errors and to increase system reliability. During the configuration, the FPGA calculates the CRC value based on the received frame data and compares it against the pre-calculated CRC value achieved at the end of the transfer. When the CRC values match, it demonstrates that there is no error in the current transfer.

The Zipwire controller accepts 32-bit data input and creates the Zipwire frame to communicate with the MCU. With the 50 MHz input clock, the PLL generates the 100 MHz system clock and 20 MHz reference clock.

In the case of the Rx controller, when the bus is in an idle state, the Zipwire signal remains low. When the Zipwire signal is driven to high, the RX controller shifts to an initial state for receiving data. During the receiving process, the Rx controller samples the received signal at a point of 80 percent of one bit length. When the LFAST sync and header fields are received with no error, the RX controller is ready to receive SIPI frame. As the SIPI frame contains SIPI header, data and CRC fields, the Rx controller interprets each field sequentially. The SIPI header contains the frame ID, channel selection and frame size. The Rx controller receives the data field according to the information of the SIPI header and calculates the CRC with the received data. When the calculated CRC value is matched to the received CRC field, the Rx controller enters the state for receiving stop bit and finishes the receiving process.

The Tx controller receives the frame ID and transmission data as inputs in order to compose a Zipwire frame. When the Tx controller receives a start signal, the Tx controller calculates the CRC-16 with the SIPI header and data to transmit. At the end of CRC operation, the Zipwire frame is generated and stored in a transmit buffer with the LFAST sync, LFAST header, SIPI header, data to transfer, CRC value, and stop bit. The Tx controller sends the Zipwire signal based on the data rate, with shifting the buffer until the transfer of the stop bit.

## 4. Results and Discussion

### 4.1. Experiment Environment and Equipment

**Figure 7:**
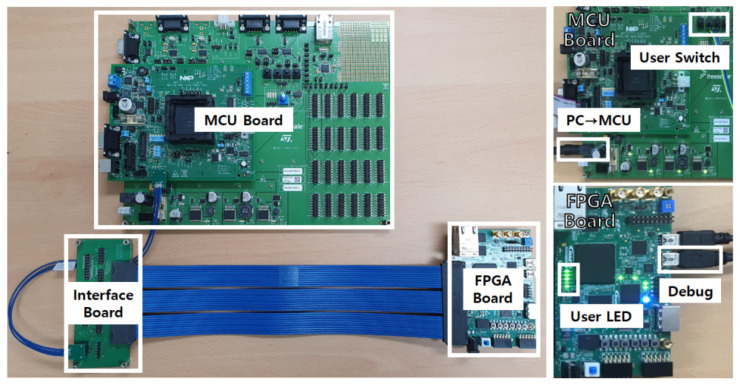
Experimental environment.

The experiment was performed with a MCU board, FPGA board, PC for external system, and interface board, as shown in Figure 7. The NXP MPC5777CEVB motherboard, which contains the 32-bit MPC5777C microcontroller, provides the software library and physical layer for Zipwire communication. In the case of the FPGA board, a Intel MAX10 FPGA development board with the 10M50DAF484C6GES FPGA was utilized. As the FPGA development board supports the LVDS physical layer through the high-speed mezzanine card (HSMC) port, the interface board was fabricated to connect the MCU board and the FPGA board. The interface board provides port compatibility and impedance matching. In this experiment, the test focused on how to recompose the FPGA-based ECU through communication with the MCU. Experiments were performed to verify functionality of the proposed system. The MCU board controls the transmission process through Zipwire communication. For Zipwire communication, the MCU and FPGA board shared two LVDS channels and one reference clock pin through the interface board. The LEDs on the FPGA board were used to confirm the reconfiguration result.

### 4.2. Experimental Results of In-System Reconfiguration

In order to demonstrate the reconfiguration of the FPGA-based ECU, the experiment was conducted. As a first step, the MCU received the trigger command and image data including reconfiguration information through the PC and stored them in the register. The MCU assigned the address and wrote the data on the memory. When the receiving process was completed, the MCU locked the memory to ensure system stability. Subsequently, the FPGA reset the address of the CFM to receive the reconfiguration data and assigned the CFM to un-protect mode to clear. The MCU provided the commands and reconfiguration data to the FPGA according to the input of the user switch. The user switches generated interrupt signals to the MCU, performing four reconfiguration processes.

1. Switch 1: Send CFM0 data to the FPGA and store in the memory.
2. Switch 2: Send CFM1/2 data to the FPGA and store in the memory.
3. Switch 3: Perform reconfiguration with CFM0.
4. Switch 4: Perform reconfiguration with CFM1/2.

When the switch 1 interrupt occurred, the trigger command was sent to the FPGA to update the CFM0 area. The FPGA received the data and swapped the data to arrange in memory. The data was written into the CFM0 and the switch 1 process was completed. The switch 2 interrupt is for updating CFM1/2 area and performed same process as the switch 1 interrupt. Switch 3 and switch 4 act as triggers for FPGA reconfiguration with the CFM0 and CFM1/2, respectively. The functionality of the system was verified by reconfiguring the FPGA with different ECU logic and the reconfiguration result was confirmed with the user LED. The results of each reconfiguration process are shown in Table 1.

In the reconfiguration process of the FPGA-based ECU, several error handlings are required for system stability. Firstly, storing error occurs when the FPGA stores the received reconfiguration data through the on-chip flash IP. The error is independent to the Zipwire communication and causes critical issues. To address the error, the MCU reads the flash data immediately after a writing operation, and compares the data to check its validity. As the on-chip flash IP stores 4-byte data with reversed bit-order at one write operation, the MCU needs to compare the data in bit unit. Secondly, disturbances which occur in the middle of the reconfiguration such as disconnection or powering-off cause errors. In order to protect the ECU from these disturbances, the ECU employs two sections of CFM which are named CFM0 and CFM1/2. One section is utilized as a default section that stores current ECU logic, and the other section stores the reconfiguration data for the update. When the reconfiguration process is performed, the MCU sets the default section to protect mode by controlling the on-chip flash IP. The FPGA restores the current logic when the errors occur in the update sequence.

Table 2 indicates the amount of FPGA resources used for the reconfigurable ECU. The designed Zipwire controller utilizes 495 logic elements, which account for less than 1% of total resources. The processor, on-chip flash IP, and dual configuration IP, which are responsible for FPGA reconfiguration utilize 9.6% of logic elements. The processor contains 64,448 memory bits to operate programming code. The system controller includes remain parts of design such as system bus. The top design occupies 12.6% of logic elements and 3.8% of memory resource in MAX10 FPGA. Therefore, the proposed ECU provides flexibility for applying more complicated ECU logic.

### 4.3. Functional Verification of Zipwire Controller

**Figure 8:**
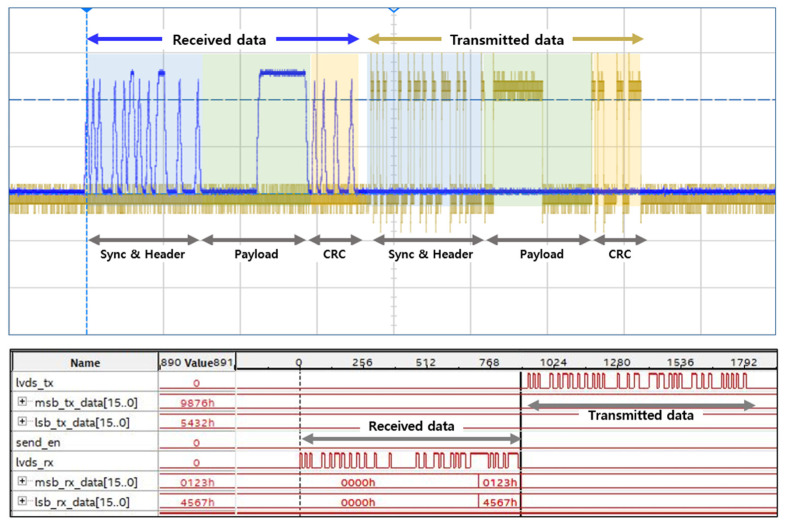
Zipwire waveform from oscilloscope and FPGA internal logic analyzer.

In order to verify the design of Zipwire controller, the oscilloscope and internal logic analyzer of the FPGA were utilized. Figure 8 depicts the waveform of the Zipwire frame captured by the oscilloscope. In this experiment, the MCU acts as a master of Zipwire communication and the Zipwire controller which is implemented on the FPGA checks the LVDS signals and sends back the response as a slave. In Figure 8, the FPGA receives the Zipwire frame that includes the sync, header, payload, and CRC field, and transmits the response with the same Zipwire frame format. The experiment confirmed that the transmission rate between the MCU and the FPGA was up to 5 Mbps and provided feasible and reliable Zipwire communication.

### 4.4. Discussion

In this work, ISR for hardware-reconfigurable ECUs was implemented with an NXP MPC5777C microcontroller and Intel MAX10 FPGA. In order to perform the reconfiguration and control the ECUs in the FPGA, a Zipwire interface was implemented with the designed Zipwire controller. The Zipwire interface ensured the reliability of communication by applying an LVDS physical layer and CRC. ISR and Zipwire communication were demonstrated throughout the experiment, and the feasibility of proposal was verified. To measure power consumption, the Power Analyzer of the Intel Quartus II 15.0 was used. The total power was estimated to be 569.26 mW, 450.70 mW for the ISR process and 118.56 mW for Zipwire communication.

For applying reconfigurable ECUs in automotive systems, the authors of [1] and [2] proposed FPGA-based ECUs. In [1], reconfiguration of the FPGA was implemented to ensure fault tolerance of the ECUs by changing the faulty design. The Xilinx Automotive Spartan-6 FPGA was employed with LogiCORE IP and XPS HWICAP IP to perform the ISR, and they achieved security and dependability in the proposed system. The authors of [2] presented the FPGA-based FlexRay communication controller, which allows sharing of resources and multiple applications through reconfiguration. They employed Xilinx Spartan 6 XC6SLX45 FPGA to implement the design.

In comparison with these papers, this work focuses on the detailed method and sequence of ISR that apply to MCU and FPGA co-designed architecture. The error sources which occur in the ISR process were analyzed. The ISR process was controlled by the MCU and FPGA to deal with errors and disturbances. The amount of FPGA resources for the proposed ECU is presented, which demonstrates the compactness of the ECU. Furthermore, by employing the MCU, the ISR enabled it to be applied to the remote system when the remote interface between the MCU and external system is implemented. Remote ISR is essential when applying hardware-reconfigurable ECUs due to the physical inconvenience of updating ECUs in a vehicle. Therefore, the proposed reconfigurable ECU achieves the advantages of application in automotive systems, and further research such as security or reliability in remote systems can be explored.

## 5. Conclusions

In this paper, a remotely hardware-reconfigurable ECU for automotive systems was proposed. The proposed system, based on in-system reconfiguration (ISR), was designed with FPGA and MCU co-designed architecture to improve system flexibility and guarantee the reliability of internal communication. In order to verify its high-speed and reliable communication, Zipwire communication was employed as an interface between the Intel MAX10 10M50DAF484C6GES FPGA and NXP MPC5777C MCU. The Zipwire controller is implemented on each board, and its functionality was verified by oscilloscope and internal logic analyzer. The proposed reconfigurable ECU was successfully implemented, and the feasibility of proposal was verified.

In future work, we plan to study and adopt methods to strengthen the security and reliability of the system. Since this works focuses on secure reconfiguration control, the experiments were conducted with compact ECU logic, utilizing less than 20% of the FPGA resources. However, as the complexity of logic and the resource utilization affect the stability of FPGA, additional experiments with complicated ECUs which include various in-vehicle networks or processing units are required. Moreover, further research is needed for the next step of remote reconfiguration of ECUs, such as reliability of the interface between external server systems and the proposed MCU. Test methodology for the updated ECUs must also meet the strict automotive standard. As security and reliability are most important factors for automotive systems, these studies can guarantee the realization feasibility of remotely hardware-reconfigurable ECUs for automotive systems.

## Figures and Tables

**Table 1:** Reconfiguration results of the FPGA-based ECU.

CFM 0 Existing Image	CFM 0 Updated Image	CFM 1/2 Existing Image	CFM 1/2 Updated Image
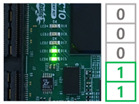	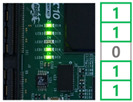	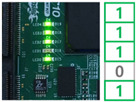	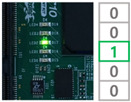

**Table 2:** MAX 10 implementation of reconfigurable ECU.

Module	Logic Elements	Memory Bits
Zipwire Controller	495 (1%)	0 (0%)
Processor	3934 (7.9%)	64,448 (3.8%)
On-chip Flash IP	591 (1.2%)	0 (0%)
Dual Configuration IP	230 (0.5%)	0 (0%)
System Controller	998 (2%)	0 (0%)
Total	6248 (12.6%)	64,448 (3.8%)
